# Electrochemical Synthesis of Carbon Quantum Dots

**DOI:** 10.1002/celc.202201104

**Published:** 2023-01-11

**Authors:** Daniele Rocco, Vyali Georgian Moldoveanu, Marta Feroci, Martina Bortolami, Fabrizio Vetica

**Affiliations:** ^1^ Department of Mechanic and Aerospace Engineering Sapienza University of Rome via Eudossiana Roma, 18 0084 Rome Italy; ^2^ Department of Chemistry Sapienza University of Rome piazzale Aldo Moro, 5 00185 Rome Italy; ^3^ Department of Basic and Applied Sciences for Engineering (SBAI) Sapienza University of Rome via Castro Laurenziano, 7 00161 Rome Italy

**Keywords:** carbon nanoparticles, carbon quantum dots, electrochemical sensors, electrochemistry, fluorescent carbon dots

## Abstract

Carbon quantum dots (CDs) are “small” carbon nanostructures with excellent photoluminescence properties, together with low‐toxicity, high biocompatibility, excellent dispersibility in water as well as organic solvents. Due to their characteristics, CDs have been studied for a plethora of applications as biosensors, luminescent probes for photodynamic and photothermal therapy, fluorescent inks and many more. Moreover, the possibility to obtain carbon dots from biomasses and/or organic waste has strongly promoted the interest in this class of carbon‐based nanoparticles, having a promising impact in the view of circular economy and sustainable processes. Within this context, electrochemistry proved to be a green, practical, and efficient method for the synthesis of high‐quality CDs, with the possibility to fine‐tune their characteristics by changing operational parameters. This review outlines the principal and most recent advances in the electrochemical synthesis of CDs, focusing on the electrochemical set‐up optimization.

## Graphical abstract

**Critical biography**: Carbon dots (CDs) are an important class of carbon nanoparticles featured by a vast range of applications, paired with low toxicity. Due to these reasons, the development of synthetic methodologies to obtain high‐quality carbon dots is of pivotal importance. This review outlines the electrochemical methodologies for the synthesis of CDs reported over the years, with a detailed analysis on how the electrochemical parameters could tune the nanodots properties.

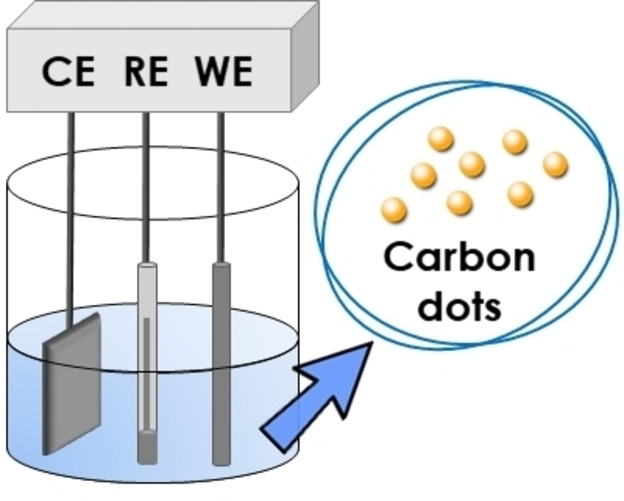

## 1 Introduction

In recent years, carbon quantum dots (CDs or CQDs) have emerged as a new, versatile, and highly applicable class of carbon‐based nanoparticles. Key features of CDs are their quasi‐spherical morphologies with sizes ranging up to 10 nm, with a graphite‐like or amorphous carbon core decorated on the surface by specific functional groups, which are directly dependent on the preparation technique and the employed starting materials. Moreover, CDs have shown peculiar properties, such as excellent photoluminescence, good photostability, high chemical inertness and excellent dispersibility in both aqueous and organic media. Pairing with these already outstanding features, CDs have gained large popularity in various research areas due to their low toxicity, excellent biocompatibility, and easy surface modification. For these reasons, CDs have found applications for in vivo imaging, energy conversion, photocatalysis, drug delivery, as well as fluorescent sensing for metal ions and pollutants.^[1]^

Furthermore, as carbon‐based structures, the starting material choice for the preparation of CDs is particularly vast, including natural‐based organic compounds, “large carbon sources” such as graphite or charcoal, but also renewable sources like biomass waste, agricultural or forestry waste or by‐products, among many others. Thus, the production of high‐value nanomaterials such as carbon dots from these resources remains of particular interest in view of sustainable chemistry and circular economy.

The two main approaches for CDs synthesis are: the bottom‐up method, starting from small organic molecules, and the top‐down approach, employing large carbon‐based materials, including biomass waste.[^1i^, ^2^] Both methods possess distinctive benefits and drawbacks, however, both approaches can be pursued by simple operations, cost‐effective and eco‐friendly techniques.

On a structural point of view, CDs can be categorized in three distinct classes: graphene quantum dots (GQDs), carbon quantum dots (CQDs) and carbonized polymer dots (CPDs). GQDs are anisotropic in nature and contain very few layers of sp^2^‐graphene fragments with sufficient numbers of surface/edge groups. CQDs, instead, are typically highly crystalline spherical sp^2^‐carbon structures. Both classes are usually obtained by top‐down methods and possess an outer surface containing principally oxygen, due to the preparation methods applied. Carbonized polymer dots, often referred to as “carbon dots”, are instead derived by bottom‐up methods. The structure of CPDs consists of an inner sp^2^‐hybridized carbon structures within sp^3^‐hybridized frameworks and abundant nitrogen/oxygen‐based surface and other molecular moieties derived directly from the substrates used due to the polymerization/carbonization process (vide infra).^[3]^

Since their discovery, the most studied CDs preparation methods rely on solvothermal or microwave‐assisted treatments of several carbon sources, both for bottom‐up and top‐down approaches. Although these developed methodologies are surely cost‐effective and industrially attractive, the monitoring of the prepared carbon dots during the synthesis is not possible. Electrochemistry can be a powerful technique for these purposes, as it allows for heterogeneous redox reactions in which the potential between two electrodes can be easily modulated and the monitoring of the passing current in the electrolytic cell could work as indicator for some of the CDs properties (vide infra). Moreover, the redox reagent is the electron: cheap, intrinsically non‐pollutant and easily dosed, hence considered a “green” reactant in chemical transformations.^[4]^

Specifically, the application of electrochemistry as intrinsically green synthetic technique[^4a^, ^4b^, ^5^] as well as the investigation of the electrocatalytic activities of CDs have been intensely studied in very recent years.

This review will outline the development of electrochemical CDs synthetic preparations over the years (2007–2022), focusing on how the electrochemical (EC) parameters, the solvent as well as the supporting electrolyte influence the morphology and fluorescence properties of the nanodots and how these can be eventually controlled. The discussion is divided according to the synthetic approach, either top‐down or bottom‐up, discussing the various starting materials used and the consequent CDs features and possible electrochemical applications.

## 2 Top‐Down

The classical approach for the synthesis of carbon nanoparticles, as above stated, consists of the exfoliation/ablation of larger carbon sources, such as graphite, graphene, polysaccharides, biomass waste, among others. These starting materials have been extensively investigated for the solvothermal and/or microwave assisted preparation of carbon dots.[^1d^, ^1e^, ^2^] Nevertheless, over the years, the electrochemical treatment of such precursors proved to be an efficient technique for the preparation of high quality top‐down carbon dots, showing a plethora of possible applications. Table 1 highlights the different methodologies reported over the years, with details on the electrochemical set‐up and parameters, the morphology and photoluminescence (PL) properties of each CDs set, as well as the eventual applications reported in the literature.

**Table 1:** Top‐down EC preparation of CDs.

Entry^[a]^	Starting material	Electrodes +/−/Reference	Solvent/ Supp. Electr.	I/E/ΔE	Size [nm]	PL exc λ_max exc_ [nm]	PL em λ_max em_ [nm]	QY *Φ* [%]	Application	Ref
1	MWCNTs	MWCNT‐carbon paper/Pt/Ag/AgClO_4_^[b]^	0.1 m TBAP in ACN	−2.0 to 2.0 V^[c]^	2.8±0.5	340	410	0.064	–	[6]
2	MWCNTs	MWCNTs coated glassy carbon/Pt foil/Pt wire (QRE)^[b]^	3.0 mm LiClO_4_ in propylene carbonate	1.1 to −1.0 V	3.0±0.3 23±2	346	456	5.1–6.3	–	[7]
3	Graphite	Pt wire/Graphite powder put in a porous plastic tube with a platinum plate/−	0.1 m TBABF_4_ in propylene carbonate	30 V	1.0–5.0	–	–	–	Pt/CDs‐coloaded graphene as catalyst for H_2_ evolution reaction	[8]
4	Graphite	GR/Pt/SCE	0.1 m NaH_2_PO_4_ in H_2_O	3 V	1.9±0.3 3.2±0.5	330 370	445 510	0.012	Possible pH indicators	[9]
5	Graphite	GR/Pt mesh/Ag/AgCl^[b]^	0.1 m PBS in H_2_O (pH=7.0)	−3.0 to 3.0 V	2.0	330	455	–	–	[10]
6	Graphene	Graphene film/Pt wire/Ag/AgCl^[b]^	0.1 m PBS in H_2_O	−3.0 to 3.0 V^[d]^	3.0–5.0	320	473	–	Co‐polymers for photovoltaics	[11]
7	Graphite	GR/GR/−	0.01 m K_2_S_2_O_8_ in H_2_O (pH=7.0)	5.0 V	3.0	500	610	1.8	Bioimaging – CDs incorporation on Hela cells	[12]
8	Graphite	GR/GR/−	0.1 m NaCl in H_2_O	9–30 V	1.76	440	523	–	–	[13]
9	Petroleum coke	Pre‐baked carbon anode/SS spiral/−	1.5 m NH_4_HCO_3_ in H_2_O	15 V	25.65	320	450	–	Copper corrosion inhibitors	[14]
10	Petroleum coke	Calcinated petroleum coke‐pitch/Calcinated petroleum coke‐pitch/−	NH_3_ in H_2_O	30 V	2.54±0.016	–	–	–	Injection pressure reduction – oil recovery	[15]
11	Carbon fibre bundles	CF/Ti mesh/−	1 m H_2_SO_4_ in H_2_O	8 V	31	385	450	–	Passivate defects in perovskite solar cell	[16]
12	Graphite	GR/GR/−	0.1 m Citric acid and 0.15–0.4 m NaOH in H_2_O	10 V	2.24–3.04	350–510	448–538	–	–	[17]
13	Graphite	GR/GR/−	0.1 m Citric acid and 0.15–0.4 m NaOH in H_2_O	8–12 V	4–15	–	–	–	–	[18]
14	Graphite	GR/GR/−	H_2_O	15–60 V	3.0–6.0	300–600	400–700	–	Photocatalysts with TiO_2_	[19]
15	Graphite	GR/GR/−	H_2_O	50 V	5.0	340	445	–	Detection of Fe^3+^	[20]
16	Graphite	GR/GR/−	H_2_O	70 V	10	340	450	–	–	[21]
17	Carbon fibres	CF bundles/Pt sheet/Ag wire (QRE)	0.1 m TBAP in ACN	2.5 V 0.5 V	2.2±0.6 3.3±0.6	300–400	400–500	1.47 1.29	–	[22]
18	Graphite	GR/GR/−	NaOH in EtOH	10–200 mAcm^−2^	1.2–3.8	365	350–800	12	Photocatalysts with TiO_2_ and SiO_2_	[23]
19	Graphite	GR/GR/Ag/AgCl	NaOH in EtOH/H_2_O	0–5.0 V	5.0	–	–	–	Electrocatalyst for O_2_ reduction	[24]
20	Graphite	GR/Pt foil/Ag/AgCl	NaOH in EtOH/H_2_O	3.0 V 7.0 V	2.9±0.3 5.2±0.6	365	438	9.5 4.6	Fe^3+^ detection and cell imaging	[25]
21	Graphite	GR/GR/−	0.1 m NaOH in EtOH/H_2_O	50 mA	7.0	365	green	–	Electrochemical detection of dopamine	[26]
22	Graphite	GR‐HOPG/Pt wire/−	BMIm‐BF_4_ and H_2_O	1.5–15.0 V	2.0–10.0	254‐260	364–440	2.8–5.2	–	[27]
23	Graphene flakes powder	GFP/GFP/−	BMIm‐BF_4_ and H_2_O (3/1)	10 V	3.0–8.0	360	450	15	Electrocatalyst for O_2_ reduction	[28]
24	Graphite	GR/GR/−	BMIm‐BF_4_ BMIm‐PF_6_ and H_2_O (4/5)	9–30 V	2.9–6.6	300–400	438–490	–	–	[29]
25	Graphite	GR/GR/−	BMIm‐BF_4_ BMIm‐PF_6_ and H_2_O	15 V	3.0–5.0	360	439	10	Fluorescent imaging of bacteria	[30]
26	Carbon fibres	CF/Pt wire/−	BMIm‐BF_4_ and H_2_O	6 V	2.4–4.2±0.5	365	435–530	0.086	Electrochemiluminescence quantification of pentachlorophenol	[31]
27	Carbon felt	GR/GR/SCE Bioelectrochemical configuration	Mineral water		5.0	500	550		H_2_ production and photocatalytic ink	[32]

[a] ACN=acetonitrile, BMIm=1‐methyl‐3‐butylimidazolium, CF=carbon fibres, GFP=graphene flakes powder, GR=graphite rod, HOPG=highly oriented pyrolytic graphite, MWCNTs=multi‐walled carbon nanotubes, PBS=phosphate buffer solution, PL=photoluminescence, QRE=quasi‐reference electrode, QY=quantum yield, SCE=standard calomel electrode, SS=stainless steel, TBABF_4_=tetrabutylammonium BF_4_, TBAP=tetrabutylammonium perchlorate. [b] In this case the electrodes refer to working/counter and reference electrode, respectively. [c] 1000 cycles. [d] 2000 cycles.

**Figure 1:**
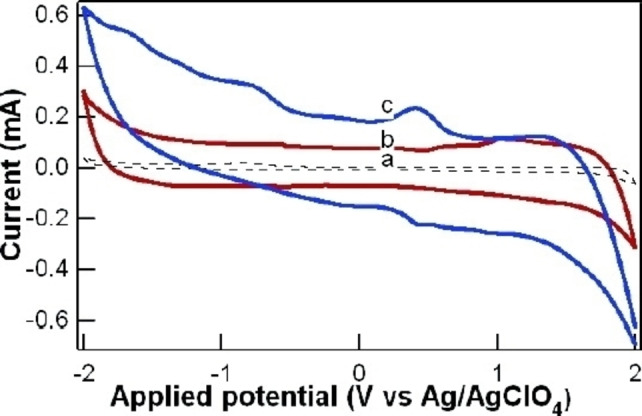
Cyclic voltammetry of carbon paper (a), pristine CNTs (b), and treated CNTs (c) in acetonitrile with 0.1mTBAP supporting electrolyte. Reproduced from ref. [6] Copyright (2007), with permission from American Chemical Society.

In 2007, the group directed by Sham, Sun and Ding reported the development of the first electrochemical synthesis of CDs by electro‐exfoliation of multi‐walled carbon nanotubes (MWCNTs) (Table 1, entry 1).^[6]^ In this work, studying the electrochemical behaviour of MWCNTs by cyclic voltammetry (CV) in degassed acetonitrile (ACN), the presence of tetrabutylammonium perchlorate (TBAP) caused the coloration of the electrolyte solution to a dark brown, with strong blue fluorescence when irradiated under UV light. After purification by evaporation and dialysis, mono dispersed CDs with average size of 2.8±0.5 nm were isolated. The electrochemical set‐up consisted in a MWCNT‐covered carbon paper used as working electrode, paired with a Pt wire and an Ag/AgClO_4_ system, as counter and reference electrodes, respectively. By applying a cycled potential between −2.0 V and 2.0 V, the authors were able to demonstrate that the synthesis of CDs was possible only in the presence of a supporting electrolyte, identifying three oxidation peaks at 0.40, 0.51 and 1.70 V, with two reduction peaks at −0.71 V and −1.68 V (Figure 1, line c).

The authors attributed these peaks to redox reactions involving CNTs defects and sidewalls. The essential presence of TBAP was explained by possible intercalation of TBA cations during the electrolysis near these defects, causing the exfoliation of CDs.

The PL properties of the obtained CDs were studied, showing a maximum of emission at 410 nm with a quantum yield (QY) of 0.064.

Following this pioneering work, a plethora of top‐down EC strategies for the preparation of CDs were reported.

On the precursors point of view, the commonly employed carbon sources are undoubtedly graphite and graphene. Nevertheless, in 2012, Pillai and co‐workers also reported the unzipping of MWCNTs into carbon dots, employing propylene carbonate (PC) as non‐aqueous solvent and LiClO_4_ as supporting electrolyte (entry 2).^[7]^ The solvent/supporting electrolyte system is reported to be essential for the formation of size‐controllable graphene CDs, by intercalation of Li^+^/PC complexes which facilitates exfoliation. The potentiostatic initial EC oxidation at 1.1 V followed by reduction at −1.0 V led to different sets of CDs, depending on the time of oxidation as well as the temperature: the higher are both temperature and oxidation time, the smaller the size of the CDs. Specifically, the optimum results were obtained performing the oxidation at 90 °C for 15 hours, yielding CDs with average sizes of 3.0±0.3 nm and higher QY than the previous example (6.3 %).

A similar technique was later used in 2020 by the group of Zhao and Jia, who also used PC as solvent in the presence of 0.1 m tetrabutylammonium tetrafluoroborate (TBABF_4_) for the exfoliation of a graphite electrode applying a voltage of 30 V in a two‐electrode cell configuration (entry 3).^[8]^ The so obtained CDs were used for the preparation of Pt/CDs‐coloaded graphene and applied as catalyst for the H_2_ evolution.

Beside these examples involving MWCNTs and propylene carbonate as solvent, as previously mentioned, graphite and graphene represent the substrates of choice for the top‐down CDs synthesis. These approaches involve the direct use of graphite rods or graphene‐containing electrodes which serve as both electrodes as well as carbon sources. Generally, water is the preferred solvent for these electrochemical processes, with the presence, but not exclusively, of a supporting electrolyte.

In 2008 the first example of EC CDs synthesis using this approach was reported by Pang and co‐workers (entry 4).^[9]^ A graphite rod (GR) was used as working electrode, paired with a Pt counter electrode and a standard calomel electrode (SCE) as reference. The effect of NaH_2_PO_4_ used as supporting electrolyte was not discussed in this case, however the PL properties of the obtained CDs were investigated in detail. Specifically, the authors highlighted that the fluorescence response of the CDs was pH‐dependent, with a maximum of emission at pH=4, and a linear decrease of the emission intensity with the increase of pH from 7 to 14, suggesting the possible application of these nanoparticles as pH indicators. Moreover, the low cytotoxicity of the synthesized CDs was demonstrated by MTT viability assay on 293T human kidney cells.

**Figure 2:**
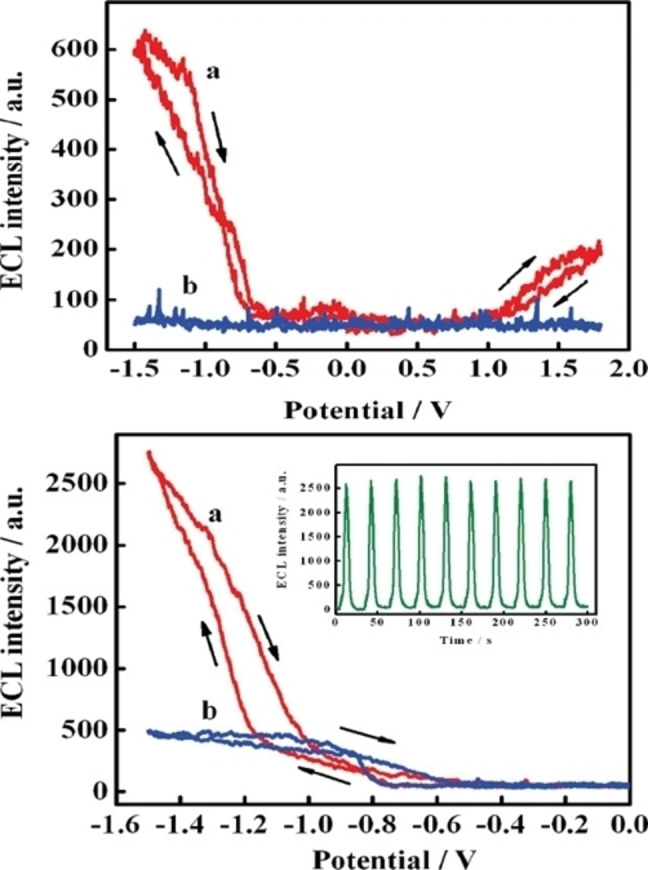
Top: electrochemiluminescence (ECL) responses (a) with and (b) without CDs at a Pt electrode in 0.1 m PBS (pH 7.0), *ν*=0.1 V/s. Bottom: ECL of CDs in aqueous 0.1 m PBS solution (pH 7.0) in the (a) presence and (b) absence of 1 mm K_2_S_2_O_8_. Inset: ECL responses of CDs/S_2_O_8_^2−^ obtained during a continuous potential scan at 0.1 V/s. Reproduced from ref. [10] Copyright (2009), with permission from American Chemical Society.

The luminescence characteristics, in particular the electrochemiluminescence (ECL) of graphite‐derived CDs were later studied in a work reported in 2009 by Chi et al (entry 5).^[10]^ Cyclic voltammetry was employed for the preparation of nanoparticles by release of water‐soluble CDs via oxidation of the graphite electrode. The applied potential at the GR electrode was cycled between −3.0 and 3.0 V in phosphate buffer solution (PBS) (pH=7). In contrast to what obtained by Ding's group^[6]^ with MWCNTs (entry 1 and Figure 1), where the charging current increased during the application of a scanning potential, the authors reported in this case a stable voltammogram, indicating presumably a different mechanism for the formation of the CDs. The authors postulated that the CDs were initially immobilized onto the porous graphite surface, and then exposed to PBS, oxidized and released in the water phase. The isolated CDs had a diameter of 2.0 nm and a maximum PL emission centred at 455 nm. Then, the ECL was initially studied with a Pt disk working electrode, with or without the prepared CDs (Figure 2, top, a and b, respectively). ECL emission was registered between −1.5 and 1.8 V, showing a maximum emission at 535 nm, indicating the promising application of ECL as monitoring technique in CDs preparation. Subsequently, the authors tested the ECL response in presence of peroxydisulfate (S_2_O_8_^2−^), a commonly used reagent in ECL, reporting a dramatic enhancement of the ECL emission intensity (Figure 2, bottom). The ECL mechanism was postulated to proceed with the formation of excited‐state CDs* via electron‐transfer annihilation of oxidized and reduced nanoparticles.

The use of PBS buffer as electrolyte solution was subsequently employed by another research group in 2011 for the synthesis of graphene quantum dots (entry 6).^[11]^ Qu and co‐workers used filtration‐formed graphene film as working electrode in a three‐electrodes EC set‐up. Monodisperse graphene CDs were obtained, with sizes ranging from 3.0 to 5.0 nm, with PL emission at 473 nm when excited at 320 nm. The XPS analysis of the composition of the prepared nanoparticles evidenced the presence of mainly hydroxyl, carbonyl, and carboxylic acids on the CDs surface. The obtained nanoparticles were integrated in a P3HT‐based solar cell, successfully enhancing the device performances.

Besides PBS and NaH_2_PO_4_ already described, other supporting electrolytes have been investigated for EC systems using water as solvent, eventually confirming the essential role of a specific electrolyte for the successful CDs preparation. For instance, in 2015 Fan and Yang reported the preparation of red‐emissive CDs by using K_2_S_2_O_8_ as supporting electrolyte (entry 7).^[12]^ A two‐electrode cell set‐up was employed with graphite rods as electrodes, applying a voltage of +5.0 V. The authors suggested that the generation of SO_4_^•−^ could be the key factor for the efficient exfoliation of CDs with a distinctive PL red emission, not common for carbon dots, which could be useful tools in cell bioimaging, as investigated by the author's cell internalization experiments. Moreover, the authors excluded the influence of the electrolyte high standard oxidative potential (*E* ^*−*^=2.01 V), since by using a stronger oxidant (K_2_FeO_4_, *E* ^*−*^=2.20 V) no similar results were obtained. Thus, they concluded that the EC generation of SO_4_^•−^ could lead to the scission of intact sp^2^ structures from graphite, hence affording extensively conjugated 3.0 nm quantum dots with a distinctive red emission and, moreover, excitation‐independent fluorescence.

In fact, a similar methodology was subsequently reported in 2019, employing a comparable cell configuration and same electrodic set‐up in water, switching the electrolyte to 0.1 m NaCl (entry 8).^[13]^ The applied voltage was investigated between 9.0 and 30.0 V, showing moderate influence on the sizes distribution (around 1.5–1.8 nm). Interestingly, the prepared CDs showed an excitation‐dependent PL emission centred between 500 and 600 nm. Additionally, a post‐oxidation of the red‐emitting CDs could fine‐change the emissive properties of the CDs from red to yellow, green, and blue, by simply controlling the reaction time at 3, 6, and 12 hours, respectively.

Nevertheless, it is clear that the choice of supporting electrolyte should be carefully considered, depending on the desired subsequent application of the carbon dots. Many authors report that alkaline conditions are necessary for an optimal CDs synthesis.

Recently, the group of Zhang reported the use of petroleum coke‐derived anodic electrode and NH_4_HCO_3_ as supporting electrolyte for the preparation of N‐doped CDs (entry 9).^[14]^ The micro‐zone electrochemical set‐up used was designed to scale‐up the synthesis of CDs, consisting of 4 cell compartments, with four anodic pre‐baked carbon anode electrodes in contact with the electrolyte solutions and a stainless steel (SS) spiral as counter electrode passing in all 4 solutions. By applying a potential of 15 V, the system afforded nitrogen‐rich CDs by degradation of the electrodes, with a high‐yield conversion of 1.87 g of CDs/g of electrode. The developed methodology yielded CDs with an average size of 25.65 nm, which were tested as copper corrosion inhibitors.

Conversely, Dai's group employed NH_3_ as supporting electrolyte in water to exfoliate petroleum coke and produced CDs with a completely different morphology (entry 10).^[15]^ The obtained CDs in this case showed a particle size averaged at 2.54 nm and were tested for injection pressure reduction and for enhancing oil recovery. Later of, two research groups screened the combination of citric acid and various concentrations of NaOH using graphite rods electrodes in a two‐electrode set‐up to produce CDs with comparable sizes (2–15 nm), by applying a voltage between 8 and 12 V (entries 12–13).[^17^, ^18^]

A different approach was instead investigated very recently by Zhou, Yuan and co‐workers, who used H_2_SO_4_ as acidic supporting electrolyte and carbon fibres bundles as working electrode and carbon source (entry 11).^[16]^ The electrochemical configuration was set‐up to adjust over time the surface of the working electrode in contact with the solution. Indeed, the immersion depth of the electrode was changed every 5 min, to compensate the gradual corrosion of the carbon fibres with the proceeding of the reaction. This technique was used to carefully control the amount of starting material in contact with the solution, avoiding the fast exfoliation of the electrode and controlling the size of the CDs to lower diameters. The so‐obtained CDs had an average size of 31 nm and were used to passivate defects in CsPbIBr_2_ perovskite solar cells.

Subsequently, several research groups envisioned the possibility to obtain top‐down CDs in pure water, without addition of additives as supporting electrolytes, by degradation of graphite electrodes (entries 14–16).[^19^, ^20^, ^21^] This approach undoubtedly reduces the environmental impact and simplifies the purification, allowing for the avoidance of the dialysis purification steps. The application of voltages between 15 and 70 V in a two‐electrode cell, monodisperse CDs were obtained with sizes ranging from 3.0 to 10.0 nm, which could be easily purified by simple centrifugation.

As previously stated, the most important goals in CDs preparation are the sizes (<10 nm) and the fluorescence properties, which can be controlled by electrochemical parameters.

**Figure 3:**
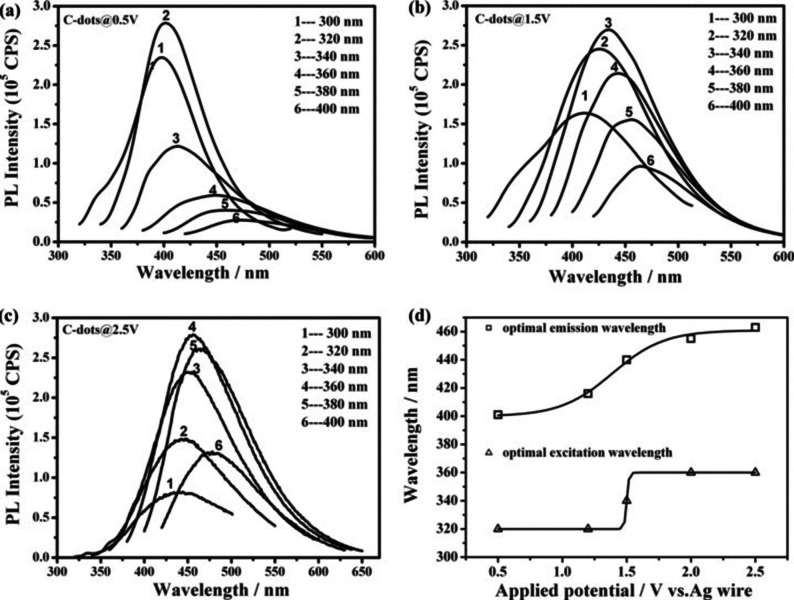
a–c) Photoluminescence spectra of C‐dots@0.5 V, C‐dots@1.5 V, and C‐dots@2.5 V. The excitation wavelengths varied from 300 to 400 nm with 20 nm increments, as indicated. d) The optimal emission and excitation wavelengths of C‐dots obtained at different potentials. Reproduced from ref. [22] Copyright (2011), with permission from WILEY‐VCH.

In 2011, Pang et al. reported a detailed morphology and fluorescence study for differently obtained CDs at various static potentials using ACN as solvent instead of water, employing 0.1 m TBAP as supporting electrolyte and carbon fibres bundles as both working electrode and carbon source (entry 17).^[22]^ The authors prepared different sets of CDs by applying a static potential from 0.5 to 2.5 V, employing a Pt wire as counter electrode and a silver wire as quasi‐reference electrode. The TEM images revealed a linear decrease of the particles diameters at the increase of the potential, from 3.3±0.6 nm at 0.5 V, to 2.2±0.6 nm at 2.5 V. These results indicated that the desired CDs diameter could be easily obtained by simple change of the potential. Moreover, since the PL properties are often related to the size of the nanoparticles, three sets of CDs (prepared at 0.5, 1.5, and 2.5 V) were studied for their fluorescence response (Figure 3).

All the obtained nanoparticles showed an excitation‐dependent fluorescence emission, with a red shift at the increase of the applied voltage. Time‐resolved photoluminescence (TRPL) analyses highlighted different behaviours for the three sets of nanoparticles, suggesting that, even if the CDs diameters were similar, morphology and recombination of electron and holes were considerably differentiated by the applied potential.

Besides ACN and water, the use of other solvents or mixtures of solvents was investigated over the years for the EC top‐down preparation of CDs.

**Figure 4:**
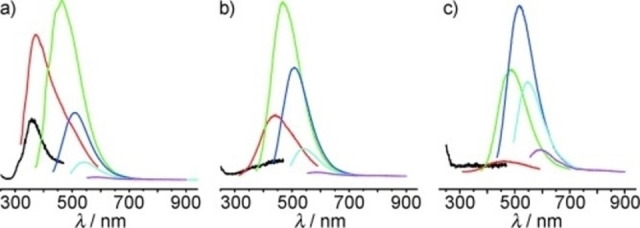
PL spectra of CDs obtained at a current density of: a) 180 mAcm^−2^ ; b) 100 mAcm^−2^; c) 20 mAcm^−2^. In these figures, the excitation wavelength for the black, red, green, blue, cyan, and pink PL lines are 240, 300, 360, 420, 500, and 580 nm, respectively. Reproduced from ref. [23] Copyright (2010), with permission from WILEY‐VCH.

The group led by Kang, Liu, and Lee envisioned the use of an electrolyte solution consisting of NaOH in pure EtOH (entry 18).^[23]^ Initially, the authors demonstrated the essential presence of an alkaline environment for the efficient production of CDs. Furthermore, operating the electrolysis of graphite rods electrodes in a two‐electrodic system at constant current, the authors studied the influence of the applied current on the PL. In Figure 4 the PL spectra at different excitation wavelengths are depicted for the CDs obtained at 180, 100, and 20 mAcm^−2^. By decreasing the applied current, a red shift of the PL emission maxima was registered. Hence, it is clear that by simply changing the applied current, it is possible to tune the PL emission of the CDs to a desired wavelength, important for an eventual application of the nanoparticles as fluorescent probes.

Subsequently, the group of Kakaei reported the use of a EtOH/H_2_O solvent system for the exfoliation of graphite rods into CDs (entry 19).^[24]^ The preparation of CDs under potentiostatic conditions was successfully developed in the presence of NaOH, which ensured the yielding of monodispersed CDs.

In the same year, Liu, Gooding and co‐workers reported the EC synthesis of CDs using the same solvent system employing a Pt foil as counter electrode and working under potentiostatic conditions (entry 20).^[25]^ Interestingly, by varying the applied potential, the authors studied the influence on the particles sizes. By operating at 3.0 and 7.0 V, the CDs measured diameters were 2.9±0.3 and 5.2±0.6 nm, respectively. These results showed an opposite trend of the influence of the applied potential compared to the work of Peng et al. previously described (entry 17), where the nanodots diameters decreased by enhancing the applied potential. The reverse trends in the two methodologies could be explained by the use of different electrolyte solutions (TBAP in ACN *vs* NaOH in EtOH/H_2_O), presumably involving different electrooxidation/exfoliation mechanisms.

**Figure 5:**
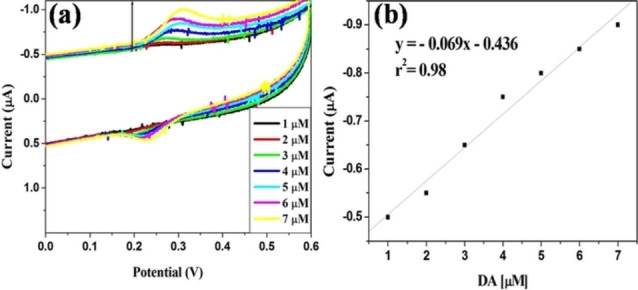
(a) CVs were recorded with different concentration of dopamine (DA) from 1–7 μM in 0.1 m PBS at SPCE/CQD modified electrode at a scan rate of 50 mV/s. (b) The corresponding calibration plot for [DA] *vs* anodic peak current. Reproduced under terms of the CC‐BY license.^[26]^ Copyright 2018, The Authors, published by ECS.

In 2018, Sundramoorthy et al. also reported the synthesis of CDs from graphite using NaOH in EtOH/H_2_O, and successfully tested the obtained CDs for the electrochemical determination of dopamine, using CDs as electrocatalysts for dopamine oxidation in PBS at pH=7.4 (entry 21).^[26]^ The obtained CDs had comparable morphologies to the ones previously prepared using the same electrolyte medium, with average diameters of 7.0 nm. The obtained nanodots were then used to modify a screen‐printed carbon electrode (SPCE) by a drop‐casting method and tested for the electrooxidation of dopamine (Figure 5). The presence of CDs efficiently enhanced the electrocatalytic activity of the SPCE electrode, increasing the current intensity registered by 13 %, with good reversibility and a linear response to the concentration of dopamine.

In the last two decades, ionic liquids (ILs) have emerged as “green” alternatives to conventional and volatile organic solvents.^[33]^ From an electrochemical point of view, they possess properties such as good ion conductivity, low viscosity, recyclability and, more importantly, a wide electrochemical potential window.^[34]^

Within this context, ILs have been also studied as solvents in the electrochemical synthesis of CDs. In 2009, Loh and co‐workers studied how different amounts of water in the IL 1‐methyl‐3‐butylimidazolium tetrafluoroborate (BMIm‐BF_4_) influenced the exfoliation mechanism and properties of CDs (entry 22).^[27]^ The presence of increasing amount of water reduced the potential window of the IL, while simultaneously shifts the onset of the exfoliation to lower activation voltages. Going from 90 % water to 10 % water, the applied voltage needed to be increased from 1.5–2.0 V to 7–8 V. Moreover, by operating in pure IL the nanodots had an average size of 2–4 nm, with a high quantum yield of 2.8–5.2 %, while instead operating at higher concentrations of water the diameters of the CDs increased to 8–10 nm. Furthermore, the authors demonstrated that using pure IL the obtained CDs were functionalized on the surface by the ionic liquid itself, which passivates the CDs surface and enhances the PL properties, presumably maximizing the dispersion properties and diminishing the quenching effects due to interparticle interactions.

**Figure 6:**
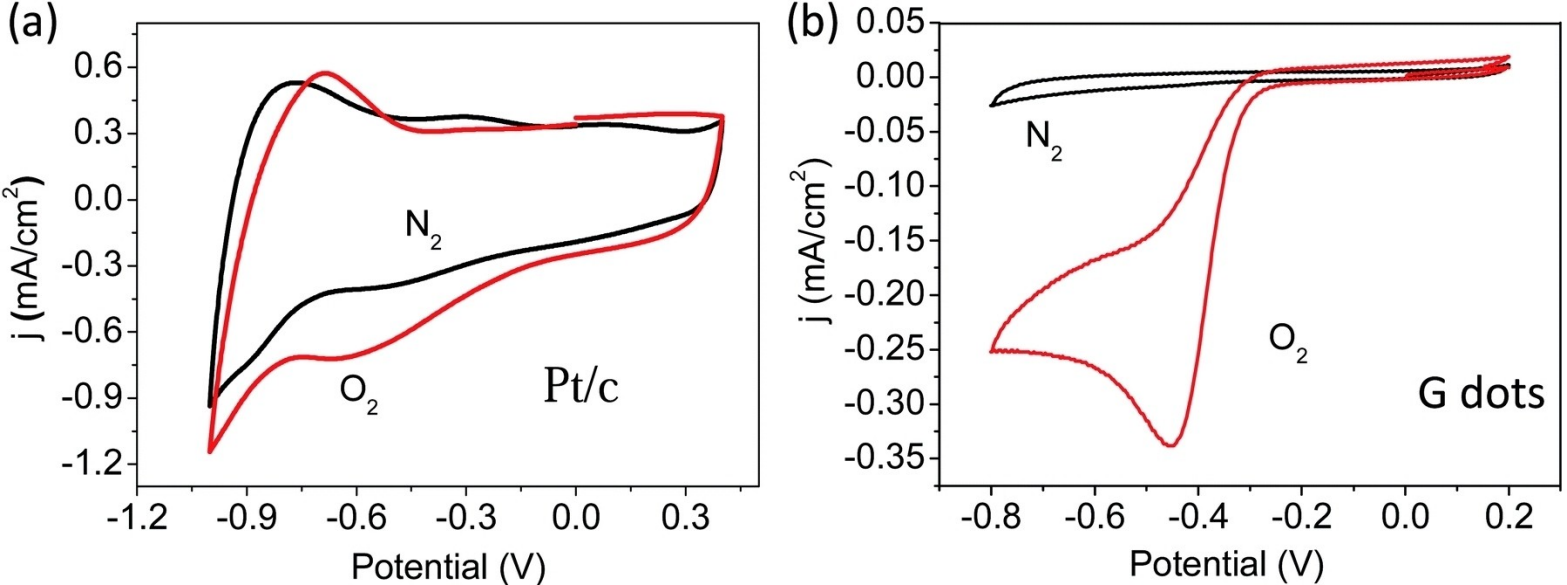
CVs of Pt/c electrode (a) and CDs on a glassy carbon (GC) electrode (b) in N_2_‐saturated 0.1 m KOH, O_2_‐saturated 0.1 m KOH. Reproduced from ref. [28] Copyright (2014), with permission from RSC Publishing.

In 2014, the mixture of BMIm‐BF_4_/H_2_O was used by the group of Ding and Wang for the synthesis of graphene quantum dots (entry 23).^[28]^ The authors set the ratio of IL/water to 3 : 1, operating with a two‐electrode cell set‐up using a graphene flakes‐modified working electrode. Also in this case, a chemical functionalization/doping of the CDs surface by oxygen and IL moieties was observed, furnishing 3.0–8.0 nm CDs with very high quantum yield of 15 %. The obtained nanodots were tested for their electrocatalytic activity in the oxygen reduction reaction (ORR), showing a 70‐time increase of the voltametric current in saturated O_2_ compared to saturated N_2_ atmosphere (Figure 6b), differently from the behaviour on commercial Pt/C electrode (Figure 6a). In fact, when the voltammetric analysis was done with Pt/C electrode, the difference in the two atmospheres was not nearly distinctive, highlighting the good electrocatalytic activity of CDs for ORR.

A similar study was later reported in 2016 by Li et al., where the effect of different amounts of water in a mixture of equimolar amounts of two ionic liquids (BMIm‐BF_4_ and BMIm‐PF_6_) were studied (entry 25).^[30]^ However, under the applied conditions, a reverse trend of amount of water/size was observed, compared to the previous example. In fact, the increasing amount of water caused this time the decrease of the particle sizes. This could be caused by the different graphite working electrode, paired with the presence of two different ionic liquids.

Additionally, Zhao and co‐workers studied the influence of the applied potential on the CDs size, using BMIm‐BF_4_/BMIm‐PF_6_/water solvent mixture (entry 24).^[29]^ Specifically, by testing an applied voltage of 9, 15, and 30 V, carbon dots with sizes of 2.9, 4.4, and 6.6 nm, respectively, were isolated. This phenomenon was explained by the authors considering the reaction time needed to consume the electrode at each potential. In fact, at low applied potential, the reaction time increased, therefore the already ablated nanoparticles in solution were longer exposed to active radicals generated during the electrolysis, which could cause the breakage into smaller particles.

Subsequently, Mao and Kun's group reported the synthesis of CDs in different BMImBF_4_/water mixture and their possible application for electrochemiluminescence quantification of pentachlorophenol (PCP) (entry 26).^[31]^

**Figure 7:**
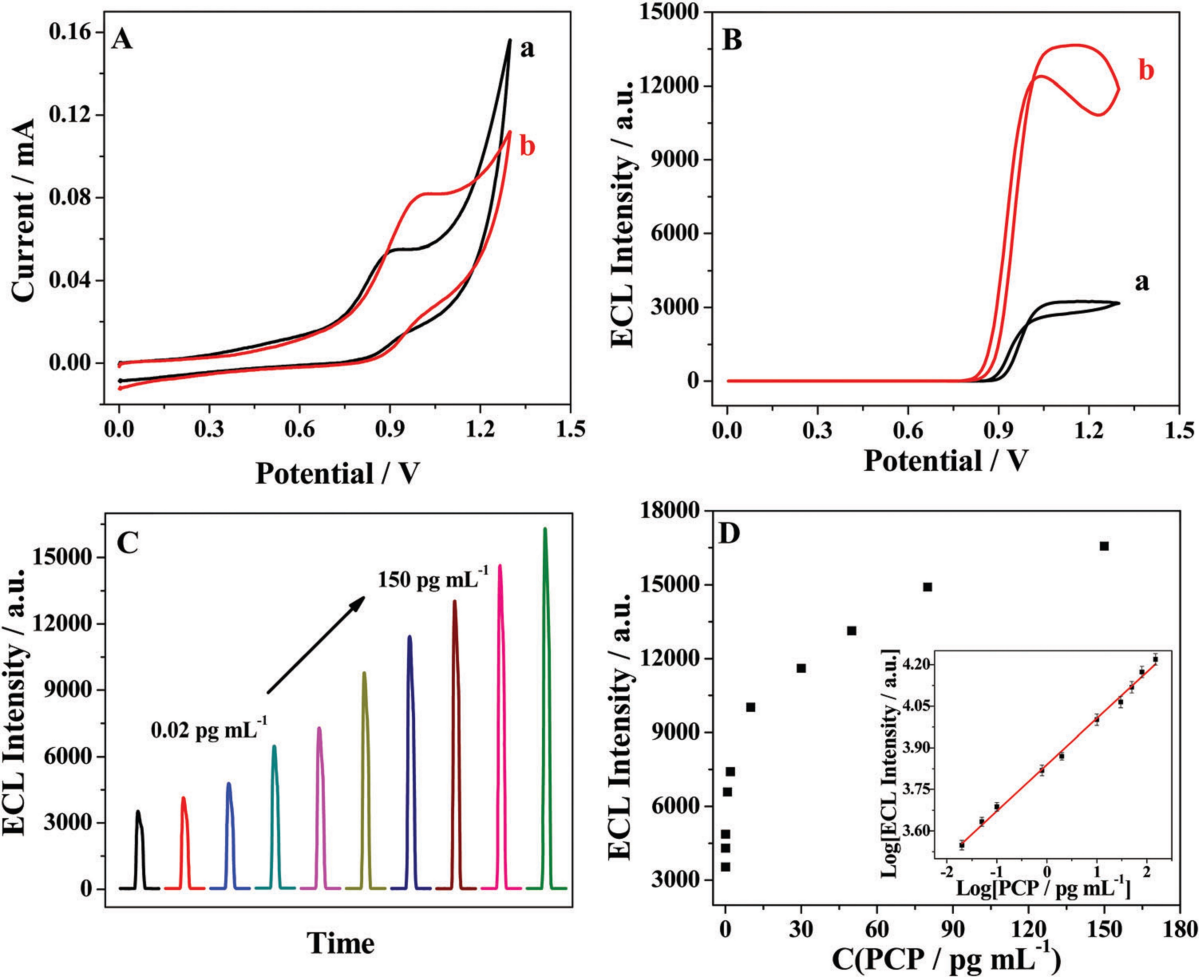
(A) CVs and (B) electrochemiluminescence (ECL) potential curves of the blue emitting CDs/Ru(bpy)_3_^2+^ system in PBS (pH 9.0) without (a) and with (b) 50 pg mL^−^1 pentachlorophenol (PCP); scan rate: 50 mV s^−1^. (C) Effect of PCP concentration on ECL behaviour of blue emitting CDs/Ru(bpy)_3_^2+^ in PBS (pH 9.0). (D) Calibration curve for PCP detection. Error bars: ±S.D., n=3. Reproduced from ref. [31] Copyright (2017), with permission from Royal Society of Chemistry.

They also studied the influence of the amount of water, showing comparable results to the work reported by Loh et al. in 2009 (entry 22). Subsequently, the authors tested the ECL behaviour of the obtained CDs in the presence of Ru(bpy)_3_^2+^, a classical ECL‐sensors reagent. In particular, in the presence of CDs, the ECL response of Ru(bpy)_3_^2+^ was considerably enhanced, allowing for the efficient electrochemiluminescence quantitative detection of PCP (Figure 7).

**Figure 8:**
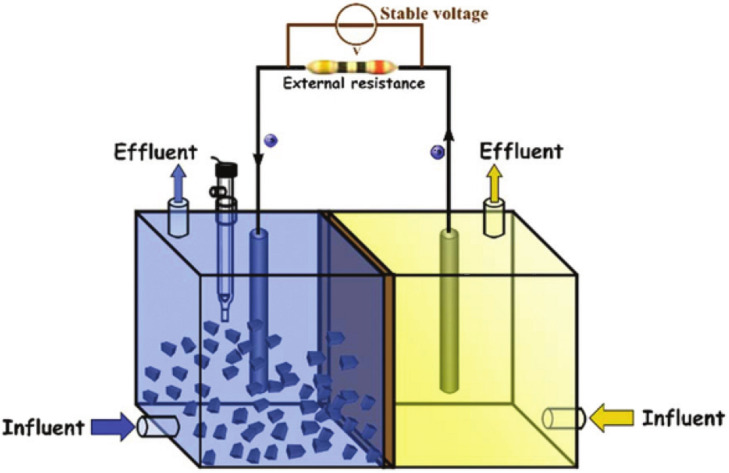
The graphical representation of CDs bioelectrochemical CDs preparation and application. Reproduced from ref. [32] Copyright (2019), with permission from RSC Pub.

Lastly, a different approach was developed in 2017. Li and co‐workers studied a bioelectrochemical set‐up for the preparation of CDs with mineral water as electrolyte (entry 27).^[32]^ The microbial fuel cell (MFC) set up consisted in a divided cell, with a proton exchange membrane, two graphite rods electrodes and carbon felt as carbon source; carrying out the electrolysis for 6 h, CDs with good photostability and an average dimension of 5.0 nm were obtained. Moreover, the presence of CDs in the MFC system enhanced the photocatalytic performances of the MFC systems in the H_2_ generation reaction (Figure 8).

## 3 Bottom‐Up EC Approach

As mentioned in the introduction section, the second approach for the preparation of CDs is the bottom‐up method, where “small” organic compounds are carbonized/oxidized/polymerized for the generation of carbon nanodots. In the last decade, the bottom‐up approach has emerged as a more efficient way to carefully control the morphology, size and PL properties of the final nanoparticles.^[2]^ Furthermore, the bottom‐up quantum dots usually possess higher quantum yields. Initially the bottom‐up technique was largely used under solvothermal conditions, nevertheless the attractiveness of the electrochemical methodologies in bottom‐up carbon dots synthesis led to subsequent investigations. The reported EC bottom‐up methods are summarized in Table 2.

**Table 2:** Bottom‐up electrochemical approaches to the synthesis of CDs.

Entry^[a]^	Starting material	Electrodes +/−/Reference	Solvent/ Supp. Electr.	I/E/ΔE	Size [nm]	PL exc. λ_max exc_ [nm]	PL em. λ_max em_ [nm]	QY *Φ* [%]	Application	Ref
1	EtOH	Pt sheet/Pt sheet/SCE	NaOH in EtOH	3.0–9.0 V	2.1–4.3	390–410	485–505	8.8–14.4	Cell imaging	[35]
2	Glycine	Pt wire/Pt wire/−	3.0 m NH_4_OH	10 V	2.4±0.4	365	440	27.1	PL detection of hemoglobin	[39]
3	Sodium citrate + Urea (1 : 3)	Pt sheet/Pt sheet/−	H_2_O	5 V	2.4	351	433	11.9	Hg^2+^ PL sensor	[40]
4	EtOH	Pt sheet/Pt sheet/−	4 m NaOH	5 V	<10.0	320–370	438–483	10.04	Fe^3+^ detection	[37]
5	ACN/BMI m‐PF_6_	Pt sheet/Pt sheet/−	BMIm‐PF_6_ in ACN (1 : 500)	15 V	3.02±0.12	355	422	13.3	Fe^3+^ detection and cell imaging	[41]
6	PrOH	Pt wire/Pt wire/Ag/AgCl	0.16 m KOH in PrOH and H_2_O	6.5 V (100 mA)	3.0–6.0	365–485	450–570	–	GC/CDs electrode for dopamine and ephedrine detection	[42]
7	EtOH	Pt foil/Pt foil/−	0.3–3.0 m NaOH in H_2_O	13 V	2.1±0.48	–	–	–	Reducing agents for core‐shell nanoparticle synthesis	[38]
8	*o*‐Phenylene diamine	Pt/Pt/−	0.1 m NaCl in H_2_O	10 V	2.8	261	570	0.47	Fe^3+^ and ascorbic acid detection	[43]
9	DTPA + EDA (1 : 3)	Pt/Pt/−	0.1 m NaOH in H_2_O	10 V	1.2	325	407	2.5	PL Detection of chlorotetracycline	[44]
10	Amino acids	Pt sheet/Pt sheet/Ag/AgCl	3.0 m NH_4_OH in H_2_O	1–10 V	2.95±0.12	270–450	400–500	7–46.2	Fe^3+^ detection and cell imaging	[45]
11	l‐ or d‐cysteine+ graphite	GR/GR/−	NaOH in H_2_O	2.0 mAcm^−2^	2.0‐4.0	390	485	11.5	Tuning laccase activity	[46]
12	l‐ or d‐glutamic acid+ graphite	GR/GR/−	NaOH in H_2_O	0.02 A	2.0‐6.0	325	405	–	Maltase inhibitor Glucose level control	[47]
13	l‐Proline or EtOH+ l‐Proline	Pt flat spiral/Pt flat spiral/SCE	NaOH in H_2_O	3.0 V	171.4	340	440	–	Heterogeneous stereoselective organocatalysts	[4e]

[a] ACN=acetonitrile, BMIm=1‐methyl‐3‐butylimidazolium, DTPA=diethylenediaminepentaacetic acid, EDA=ethylenediamine, GC=glassy carbon, GR=graphite rod, PL=photoluminescence, QY=quantum yield, SCE=standard calomel electrode.

In 2014, Zhang et al. reported the first EC bottom‐up approach using simply EtOH as carbon source (Table 2, entry 1).^[35]^ By using a two Pt spirals as working and counter electrodes and a SCE as reference, the authors tested different applied potentials for the electrooxidation of EtOH in the presence of NaOH. Initially they proved the importance of alkaline conditions, based on control experiments in H_2_SO_4_ or Na_2_SO_4_ as supporting electrolytes, where the production of CDs was unsuccessful. Instead, Na_2_HPO_4_ or Na_2_CO_3_, beside NaOH, afforded monodisperse CDs, proving the essential presence of HO^−^ to efficiently electrooxidize the alcohol into nanoparticles.^[36]^ The proposed mechanism involves electrooxidation of EtOH, cross‐linking reactions and subsequent dehydration steps. The techniques allowed for the preparation of high oxygen‐containing nanodots, with mainly alcoholic functional groups on the active surface. Furthermore, a detailed study on the influence of the applied potential was undertaken. By applying 3.0, 4.5, 6.0, and 7.5 V and carrying out the electrolysis for 4–5 hours, CDs with average sizes of 2.1, 2.9, 3.5 and 4.3 nm were isolated, respectively, proving the proportional increase of dimension with the enhancement of the applied voltage. Since normally the PL properties of CDs are influenced by the size and morphology, the four sets of CDs had also different fluorescence behaviours. Higher dimeter nanoparticles registered a red shift in the maximum wavelength emission, going from 400 to 600 nm, in all cases with excitation‐dependent photoluminescence. Subsequently other research groups used EtOH as carbon source, with similar EC cell configurations involving NaOH as supporting electrolytes, yielding comparable results in terms of both morphology and PL properties, applying the obtained CDs as fluorescence detectors for Fe^3+[37]^ and as reducing agents for the synthesis of core‐shell nanoparticles^[38]^ (entries 4 and 7, respectively).

In the same year, Chang and co‐workers envisioned the use of another readily available carbon source for the production of CDs: glycine (entry 2).^[39]^

The use of naturally derived amino acids would in fact be economically favourable and the possibility to change the amino acids could lead to libraries of readily available CDs, whose properties and ranges of applications could be completely different. In this work, by applying a voltage of 10 V, glycine molecules were electrochemically oxidized to iminium ions, which reacted with other glycine via amidation reaction. Subsequently, electropolymerization, carbonization and passivation steps afforded fluorescent carbon nanodots with a yield of ca. 55 %. The obtained CDs had 2.4±0.4 size, with an apparent crystalline structure. Moreover, the measured quantum yield was 27.1 %, which is higher than any other EC‐derived CDs prepared up to that year. The effect of NH_4_OH as supporting electrolyte was identified as both N‐doping and passivation agent, coupled with the maintaining of the necessary alkaline environment. Nevertheless, the amount of NH_4_OH needed to be lower than 3.0 M, not to reduce the reactivity of glycine in the amidation reaction step. Also in this case, performing the reaction at higher potentials led to an increase of the sizes paired with a red shift in fluorescence emissions.

Afterwards, Zhang's group selected citric acid and urea as carbon sources (entry 3).^[40]^ Indeed, these two components have been classically used to prepare CDs under solvothermal and/or microwave‐assisted methods. The authors envisioned the use of different mixtures of citric acid and urea in pure water under a potentiostatic electrochemical set‐up. In fact, the two substrates functioned as both carbon sources and supporting electrolytes, allowing for the avoidance of additional additives. The optimum citric acid/urea ratio was set at 1 : 3, affording CDs with a quantum yield of 11.9 %, and average sizes of 2.4 nm.

In 2016, Xu and Liu investigated the application of ionic liquids as supporting electrolytes and ACN in bottom‐up preparation of nanodots. In particular, the influence of BMIm‐PF_6_/ACN ratio as well as the applied potential on the CDs features was studied (entry 5).^[41]^ A higher starting current and a shorter reaction time were observed with the increase of the amount of IL, indicating an IL‐assisted electrooxidation/cross‐linking/dehydration mechanism. Moreover, the QY also increased with enhanced amounts of IL, presumably due to doping processes from the ionic liquid onto the CDs surface. The methodological study dependent on the applied voltage, using the optimum IL/ACN ratio of 1 : 500, highlighted that 15 V were needed to guarantee good PL properties paired with reasonable reaction times, since the reducing to 5 V led to the increase of reaction time of at least 3 days, due to the lower passing current.

**Figure 9:**
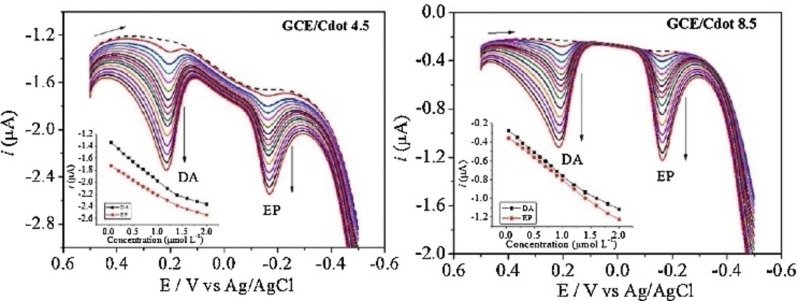
Simultaneous determination of epinephrine and dopamine using the a) GCE/C‐dots 4.5 (4.5 h reaction time) and b) GCE/C‐dots 8.5 (8.5 h reaction time), in PBS 7.0. Reproduced from ref. [42] Copyright (2016), with permission from Elsevier.

In the same year, a detailed chronoamperometrically‐controlled synthesis of PrOH‐derived CDs was reported by the group of Canevari and Toma (entry 6).^[42]^ Using two Pt wires as working and counter electrodes and a Ag/AgCl reference electrode and working under an applied constant potential of 6.5 V and a basic KOH electrolyte solution. The obtained CDs were monodispersed, with a crystalline structure and no indications of agglomerates formation. Carrying out the electrolysis for higher reaction time (8.5 h) the sizes of the nanoparticles increased. This result is opposite to what usually obtained in top‐down methods (see previous section). In fact, when performing the synthesis by exfoliation, initially bigger particles are released from the electrode and subsequently, in the presence of reactive radical species, they are cut‐down to smaller particles. When using small molecules as substrates, the constant presence of substrate and radicals for longer times causes the continuation of cross‐linking/dehydration reaction, accreting the nanodots to higher diameters. The obtained CDs were subsequently used to modify a glassy carbon electrode (GCE) for the simultaneous quantification of epinephrine and dopamine by differential pulse voltammetry (Figure 9). In fact, epinephrine and dopamine showed separated potential responses, both with a concentration‐dependent linearity, allowing for their simultaneous detection, with a LOD in the micromolar scale.

Other useful starting materials in CDs synthesis are organic amines. In fact, since in electrochemical preparations an alkaline environment seems crucial, organic amines ensure the basic pH while being at the same time a carbon source.

Xu and co‐workers reported the use of *o*‐phenylenediamine (OPD) as precursor for the synthesis of CDs with a high Stoke's fluorescence shift (entry 8).^[43]^ A two‐electrode set‐up was used under potentiostatic conditions, in water, affording 2.8 nm nanodots, with good photoluminescence properties and an unprecedented Stoke's shift of 309 nm. The authors tested the *meta* and *para*‐substituted diamines as well, obtaining monodisperse carbon dots; however, the Stoke's fluorescence shifts were not that pronounced (126 nm and 122 nm, respectively). They postulated that the *ortho* functionalization allowed for the generation of a higher largely conjugated system, thus providing a higher shift between PL excitation and emission maxima.

More recently, Chiu et al. proposed the use of diethylenediaminepentaacetic acid (DTPA) and ethylenediamine (EDA) for the synthesis of CDs under a similar electrochemical two‐electrode set‐up (entry 9).^[44]^ They optimized the ratio between the substrates, obtaining the best results in terms of QY with DTPA/EDA 1 : 3.

**Figure 10:**
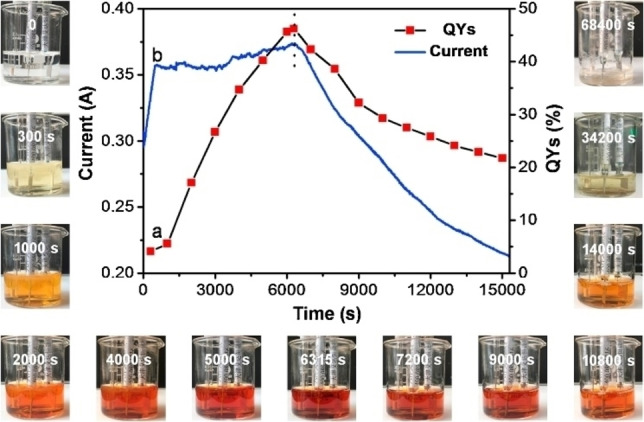
(a) The amperometric *i*‐*t* curve at applied potential of 3 V and (b) corresponding QYs evaluated at different reaction time for the N‐CQDs generation using a three‐electrode electrochemical/electroanalytical (ECA) system. Photographs surrounding the curves are collected for the reaction mixtures at different time periods during the ECA generation process, respectively. Electrolyte component: 0.5 m Asp solution in the presence of 3 m NH_3⋅_H_2_O. Reproduced from ref. [45] Copyright (2017), with permission from Elsevier.

**Figure 11:**
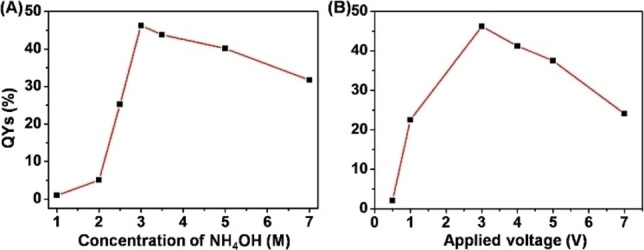
The correlation between QYs of N‐CQDs and (A) the concentration of NH_3⋅_H_2_O or (B) the applied voltage. Reproduced from ref. [45] Copyright (2017), with permission from Elsevier.

**Figure 12:**
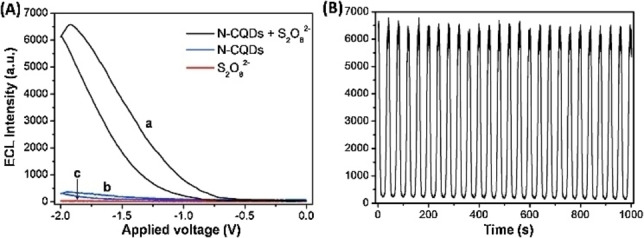
(A) ECL intensity‐potential curves of 100 mg mL^−1^ Asp‐N‐CQDs in 100 mm PBS (pH 9.0) (a) with and (b) without 10 mm K_2_S_2_O_8_, (c) 10 mm K_2_S_2_O_8_ alone. The scan rate is 100 mV s^−1^. (B) ECL intensity‐time curve under continuous CV scanning for 1000 s (25 cycles) for Asp‐N‐CQDs/K_2_S_2_O_8_ system. Reproduced from ref. [45] Copyright (2017), with permission from Elsevier.

In 2017, Xu and Liu reported a more detailed study on the use of several amino acids as substrates in an electrochemical synthesis of CDs, where the optimal quantum yield could be controlled by the maximum current value on the amperometric current/time curve during the EC process (entry 10).^[45]^ Initially they studied aspartic acid as substrate, using ammonium hydroxide as electrolyte solution. By plotting the measured quantum yields of CDs obtained at different times, the authors identified that the maximum of registered passing current during the electrolysis corresponded to the maximum QY on the resultant CDs (Figure 10). This finding evidenced the possible monitoring of the quality of the CDs during the electrochemical preparation, by simply measuring the current. Subsequently, the effects of the amount of NH_4_OH as well as the applied voltage on the nanodots QYs was investigated, optimizing the reaction conditions (Figure 11). Lastly, the ECL properties of the prepared CDs were studied, cycling the potential between −2.0 and 0 V. The signals of the pure CDs were fairly low, nevertheless, in the presence of the classical ECL co‐reactant S_2_O_8_^2−^, the ECL signal was considerably intense, suggesting stable ECL properties of the analyzed CDs and envisioning a broader scope of applications of the so‐prepared nanodots (Figure 12).

Chirality is undoubtedly a fundamental property of nature, ranging from small organic molecules to solid objects, as well as to nanostructures. Due to homochirality of biological chemical components, the different interactions on optically active molecules in a chiral environment are essential for numerous applications. Thus, the study of chiral interactions and the synthesis of optically pure organic compounds remains a key challenge in modern organic chemistry. In the realm of CDs chemistry, the chirality aspects of these nanostructures have been less explored. It has been demonstrated the possibility of achieving chiral carbon dots (CCDs) via either late‐stage surface modifications or with one‐pot preparations starting from chiral molecules under solvothermal conditions.^[48]^ In fact, the chiral information of the starting material could be retained during the formation of the core, being predominantly present on the surface.

Within this context, also electrochemical approaches to bottom‐up carbon dots synthesis focused the attention on the chirality aspect.

In 2018, Kang and co‐workers studied the synthesis of CDs starting from the natural amino acid l‐cysteine, in a similar strategy used for other amino acids before, working under galvanostatic conditions (entry 11).^[46]^ However, the authors realized that the produced CDs retained the chiral information, as proved by the electronic circular dichroism spectrum. Later on, the authors prepared another set of CDs starting from the unnatural d‐cysteine, providing optically opposite CDs, as predicted. Afterwards, the biocatalytic activity of these two sets of nanodots onto the enzyme laccase activity was tested. Due to the opposite optical nature, paired with a chiral enzyme, the capability of the CDs to tune laccase activity was substantially different. Specifically, l‐CDs could enhance the activity of the enzyme, while the d‐CDs acted as a non‐competitive inhibitor, proving that the enantiosynthesis of CDs could enlarge the range of bio‐applicability of carbon quantum dots. The subsequent year, Kang's group applied the same electrochemical synthesis to prepare l‐ or d‐glutamic acid CDs sets, which differently tuned the activity of the enzyme maltase for glucose level control (entry 12).^[47]^

Recently, our group studied as well the EC synthesis of chiral CDs to be applied as chiral nano‐organocatalysts (entry 13).^[4e]^ Since in chiral carbon dots amino acidic moieties are preserved onto the active surface, and that amino acids‐derivatives are classical organocatalysts used to perform stereoselective reactions, we envisioned the preparation of CDs directly from l‐proline to prepare heterogeneous nano‐organocatalysts. A detailed methodological investigation of the electrochemical set‐ups, ranging from galvanostatic to potentiostatic conditions, as well as the practical use of a simple (two electrodes) power supply, resulted in high diameter‐CDs, with not optimal catalytic performances. However, the electrochemical synthesis of EtOH‐derived CDs, followed by a late‐stage chemical functionalization of the active surface with l‐proline led to efficiently active CDs, which were applied to a stereoselective aldol reaction in DMSO/H_2_O solvent mixture, obtaining high yields as well as very high enantiomeric excesses. As an additional advantage, the CDs were successfully reused for subsequent reaction cycles, by simply removing the products via extraction.

## 4 Summary and Outlook

In conclusion, in this review we wanted to depict the various electrochemical methods reported over the years for the preparation of carbon dots (CDs). Scope of this review was also to put in evidence how the electrochemical set‐up and carbon precursors choice could influence the properties and ranges of applications of the generated nanodots. Moreover, this review focusses the attention on the effect that the electrolyte solution, such as an ionic liquid, could have on the outcome of the nanodots properties, potentially varying the formation mechanism and consequently the morphology and chemical structures of CDs. In particular, the operational simplicity of electrochemical procedures as well as the easy control over parameters, such as applied voltage or current, offers a valuable tool to fine tune the characteristics of the CDs for the needed envisioned application, resulting in a valuable and versatile technique.

Moreover, the electrochemical equipment, once intended only for specialized laboratories, is now affordable to all organic chemistry laboratories, both from the cost point of view and from the operation simplicity one. This, along with the boost to the use of electricity in the chemical synthesis by the European Union and other worldwide organizations, should induce also non‐electrochemical scientists to test this versatile technique.

Future exploitations of the electrochemical methodology applied to the bottom up synthesis of nanostructures will surely go also in the direction of a simultaneous synthesis and functionalization of surfaces with such carbon dots, in both an achiral or chiral fashion, in order to obtain heterogeneous catalysts to be easily removed from the reaction mixture. Moreover, possibly (when a conductive substrate is functionalized) a nanostructure chiral electrode could be obtained, which could boost chiral electroorganic synthesis, at this stage still understudied.

We can conclude that the use of electrochemical methods for CDs synthesis, especially for the most recent chirality involvement in the nanodots world, could pave the way to future advances in green chemistry, leading to more sustainable approaches in organic chemistry, medicinal chemistry and sensing applications.

## Conflict of interest

The authors declare no conflict of interest.

5

## Biographical Information

*Daniele Rocco obtained his Master's degree in Chemistry at the “Sapienza” University of Rome (Italy) in 2017. In the same University, he obtained his doctorate at the Department of “Ingegneria Astronautica, Elettrica ed Energetica”. His research activity is focused on the design, synthesis and characterization of new molecules in the field of Organic Electronics and on organic electrosynthesis and flow electrochemistry. He is currently Assistant Professor in the Department of Mechanical and Aerospace Engineering at Sapienza University*.

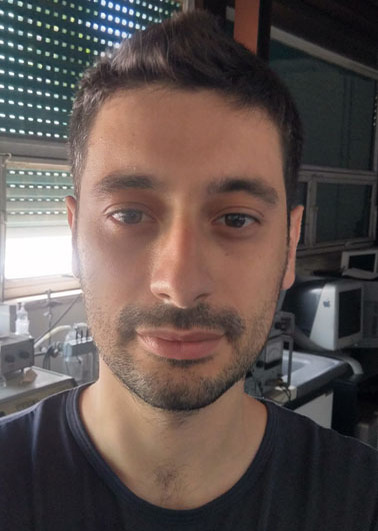

## Biographical Information

*Vyali Georgian Moldoveanu was born in 1997 in Romania. He studied at “Sapienza” University of Rome where he achieved his Master Degree in Organic and Biomolecular Chemistry in 2022. During his master he worked on domino reactions, studying 1,3‐dipolar cycloadditions that led to the synthesis of spiropyrrolizidin‐oxindoles. He is currently pursuing his PhD under the supervision of Dr. Fabrizio Vetica. His research interest includes enantioselective organocatalytic domino/cascade reactions for the synthesis of heterocyclic compounds*.

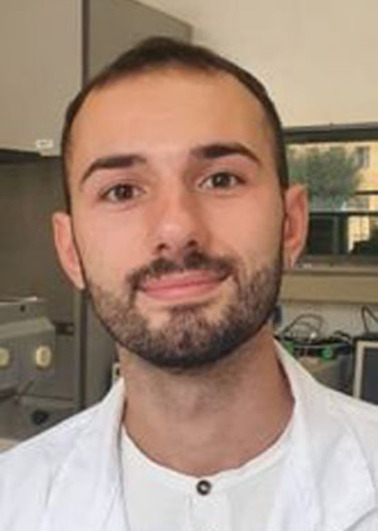

## Biographical Information

*Marta Feroci, graduated in Organic Chemistry, received her Ph.D. in Organic Chemistry in 1994 from Sapienza University of Rome. She is full professor of Chemistry for Engineering at Sapienza University. Her research interests are mainly in the field of electroorganic synthesis and classical organic synthesis. In particular, the generation of electrogenerated bases and nucleophiles by cathodic reduction in both classical organic solvents and in ionic liquids (with NHC generation) and their use in synthesis and organocatalysis*.

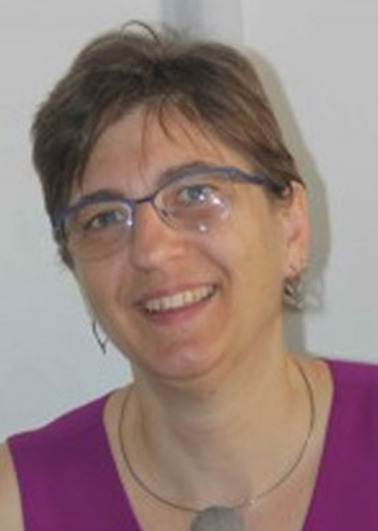

## Biographical Information

*Martina Bortolami obtained her Master's degree in Pharmacy at Sapienza University of Rome, Italy, in 2016. In the same University, at the Department of “Chemistry and Technology of Drug”, she obtained her PhD in Pharmaceutical Sciences in 2019. Afterwards, she worked as Postdoctoral Researcher on organic and electro‐organic synthesis of molecules of industrial and pharmaceutical interest at the Department of “Basic and Applied Sciences for Engineering” of Sapienza University of Rome. Her research activity is focused on the organic electrosynthesis, on the design, synthesis and characterization of new molecules in the field of pharmaceutical chemistry as well as the synthesis of electrochemical synthesis of carbon dots for catalytic applications*.

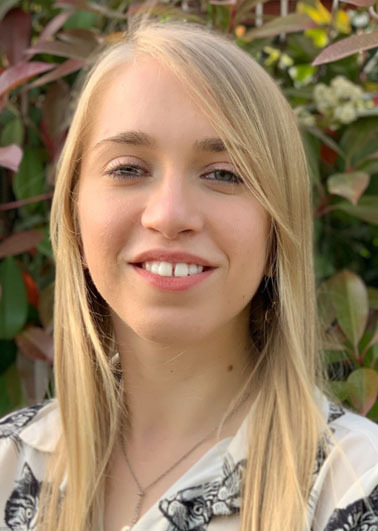

## Biographical Information

*Fabrizio Vetica received his Master Degree in Organic Chemistry at “Sapienza” University of Rome. Afterwards, he obtained his Ph.D. in Organic Chemistry in 2018 under the supervision of Prof. Dieter Enders at RWTH Aachen University. Subsequently, he worked as Labteam leader at BASF SE working on heterogeneous catalysis. In 2019 he joined ISOF‐CNR in Bologna as Postdoc. Since 2020 he is Researcher ‐ Assistant Professor of Organic Chemistry in the Department of Chemistry of Sapienza University of Rome. His research interests focus on sustainable organic chemistry: asymmetric organocatalysis, natural products synthesis, electroorganic synthesis and synthesis of carbon dots for catalytic and biomedical applications*.

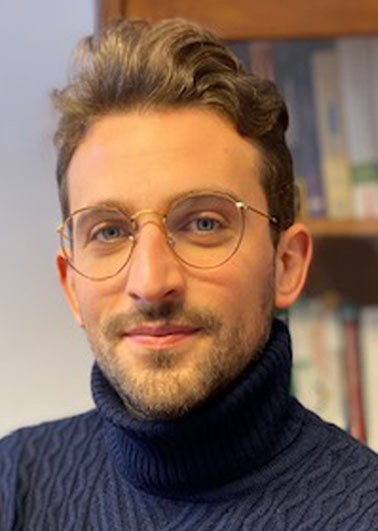

## Data Availability

Data sharing is not applicable to this article as no new data were created or analyzed in this study.
